# Recent advances in date palm genomics: A comprehensive review

**DOI:** 10.3389/fgene.2022.959266

**Published:** 2022-09-13

**Authors:** Hifzur Rahman, Prashant Vikram, Zied Hammami, Rakesh Kumar Singh

**Affiliations:** International Center for Biosaline Agriculture (ICBA), Dubai, United Arab Emirates

**Keywords:** date palm, genomics, diversity, molecular markers, transcriptomics

## Abstract

As one of the oldest fruit trees of the Arabian peninsula, other Middle-Eastern countries, and also North Africa, the date palm (*Phoenix dactylifera* L.), is highly significant for the economy of the region. Listed as part of UNESCO’s Intangible Cultural Heritage of Humanity, the date palm is believed to be the first tree cultivated by human beings, and was probably first harvested for its fruit nearly 7,000 years ago. Initial research efforts in date palm genetics focused on understanding the genetic diversity of date palm germplasm collections and its phylogenetic history, both important prerequisites for plant improvement. Despite various efforts, the center of origin of the date palm is still unclear, although genomic studies suggest two probable domestication events: one in the Middle East and the other in North Africa, with two separate gene pools. The current review covers studies related to omics analyses that have sought to decipher the present genetic diversity of the date palm. With advances and cost reductions in sequencing technologies, rapid progress has been made in the past few years in date palm genomics research. Along with organellar genomes, several reference genomes of the date palm are now available. In addition, several genotypes have been re-sequenced, either to detect single nucleotide polymorphisms (SNPs), or to study domestication and identification of key genes/loci associated with important agronomic traits, such as sex, fruit color, and sugar composition. These genomics research progress has paved the way to perform fast-track and precise germplasm improvement processes in date palm. In this study, we review the advances made in the genetics and genomics of the date palm so as to strategize targeted crop improvement plans for marginal areas of the Middle Eastern peninsula, North Africa, and other parts of the world.

## Introduction

Date palm (*Phoenix dactylifera* L.) is one of the oldest fruit trees in the Arabian Peninsula (AP), other countries of the Middle East, and the arid regions of North Africa. Its origin is not yet known; however, evidence indicates this was possibly near Iraq. In the AP, the date palm holds cultural importance for the people, besides being a critically important staple food and a major source of income. It is known for multiple products and purposes, including fruit, fiber, fuel, and sheltering material. In addition to providing calories, dates serve as a source of vitamins and minerals (El Hadrami and Al-Khayri, 2012), rendering them a healthy and nutritious calorie option. The global trade in dates was valued at about USD $1.2 billion in 2016, thereby contributing to the livelihood and income of millions of rural smallholders in the AP and surrounding areas [FAO: Microsoft Word - Conference-side-event-Dates-Saudi-Arabia.docx (fao.org)]. Globally, date palms are cultivated on 1.1 million hectares of land with a production of about 8.7 million tons (FAOSTAT, 2018). Iran, Algeria, Iraq, Saudi Arabia, and Egypt share 59% of the total harvested area and 66.5% of the total production, with maximum yield coming from Egypt (Table 1) (FAOSTAT, 2020). The area under date palm cultivation has also increased continuously during recent decades because of the crop’s adaptability to the harsh climate.

**TABLE 1:** Cultivar diversity and production indices across major date palm producing countries.

Country	Cultivar evaluated^a^	Area harvested (ha)^b^	Yield (kg/ha)^b^	Production (tons)^b^
Algeria	1,000	170,500	6756.1	1,151,909
Egypt	52	50,834	33264.3	1,690,959
Iraq	400	245,033	3001.0	735,353
Iran	400	154,145	8326.6	1,283,499
Libya	95	32,868	5404.3	177,629
Morocco	453	61,332	2334.2	143,160
Oman	250	25,630	14380.7	368,577
Pakistan	NA	106,488	5101.7	543,269
Saudi Arabia	450	152,705	10096.4	1,541,769
Sudan	400	37,000	12576.3	465,323
Tunisia	250	72,205	4598.0	332,000
Yemen	321	15,038	4627.6	69,590
United Arab Emirates	120	38,422	8554.2	328,669

a Abul-Soad et al. (2017).

b Data from FAOSTAT (2020).

Despite being one of the most successful fruit crops in arid, semiarid, tropical, and subtropical regions, there has been relatively little research into the genetics and molecular genetics of the date palm compared to other commercial fruit trees. Genomics approaches are an exception here, and rapid advances have been made in the past decade. So far, the date palm genome, along with its organellar genomes, has been sequenced (Al-Dous et al., 2011; Fang et al., 2012; Khan et al., 2012; Al-Mssallem et al., 2013; Asaf et al., 2018). Several genotypes have been re-sequenced, either to detect single nucleotide polymorphisms (SNPs) (Thareja et al., 2018), or to study domestication and marker-trait association (Hazzouri et al., 2015, 2019; Gros-Balthazard et al., 2017). In addition to SNPs, other marker resources have been used in the past for diversity studies and the identification of cultivars, including random amplified polymorphic DNA (RAPD), inter simple sequence repeats (ISSR), simple sequence repeats (SSR), and amplified fragment length polymorphisms (AFLP), etc. Early sex deteremnation is an important trait in the date palm. Because the date palm is dioecious in nature, efforts have been made to develop specific markers for early detection of the female plant. Molecular markers have also been developed for brittle leaf disease (BLD) resistance in the tree. In the recent past, red palm weevil (*Rhynchophorus ferrugineus* Olivier) has had a devastating effect on date palm yields in the Arabian Peninsula (Kassem et al., 2020). At the International Center for Biosaline Agriculture (ICBA), UAE, efforts are underway to address this biotic stress with the help of advanced genomics tools (ICBA, unpublished).

Although there has been little progress in genomics applications in date palm improvement, this study represents an effort to review what progress has been made, as well as identify the future prospects for date palm genomics, given the importance of this crop for the livelihood of vast numbers of farmers in the AP.

## Botanical description of date palm

**FIGURE 1:**
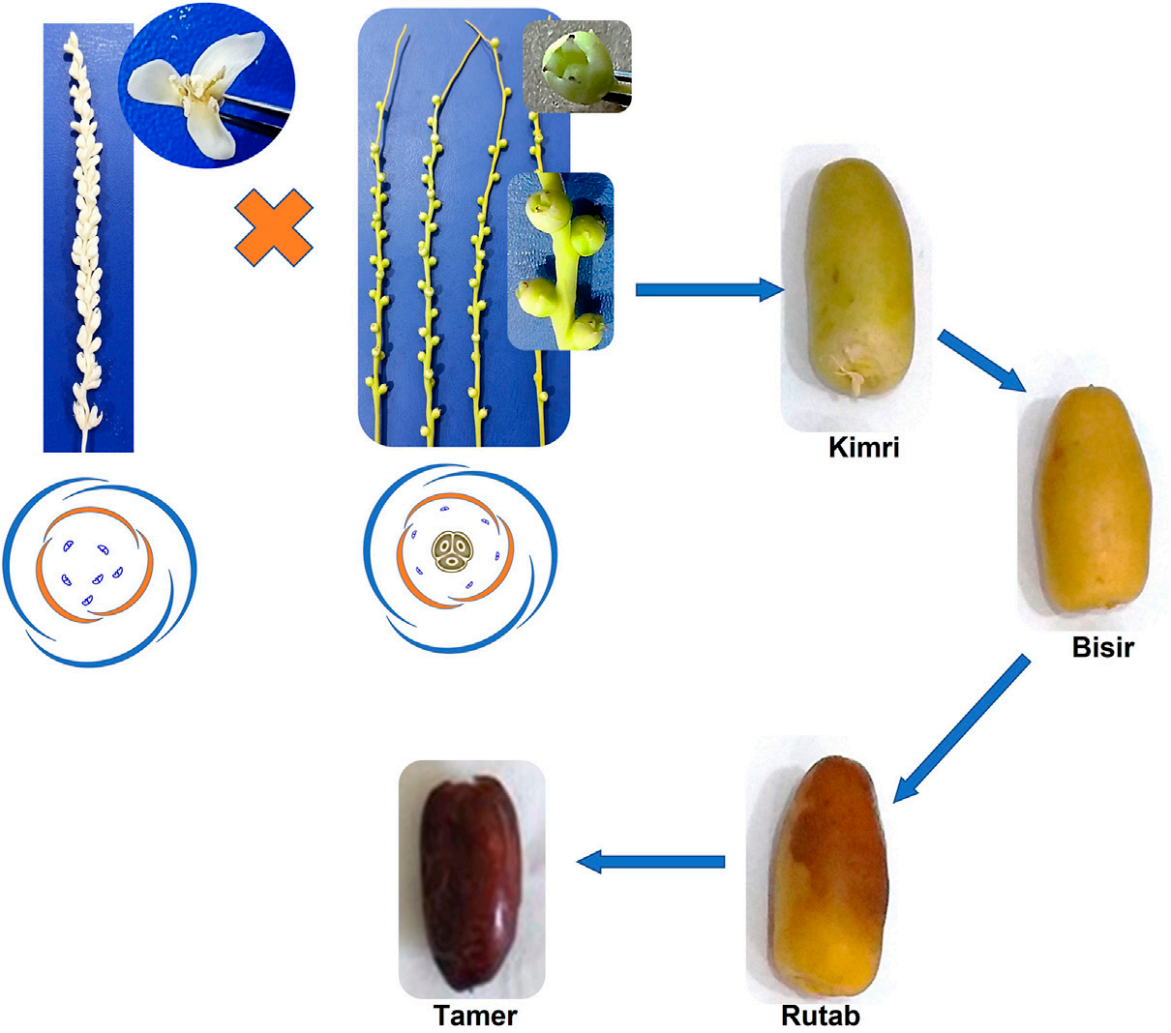
Floral biology and developmental stages of the date palm fruit.

The date palm (*Phoenix dactylifera* L.) is a perennial monocotyledonous plant belonging to the family Arecaceae (Palmae). Mature date palm plants are the tallest among *Phoenix* spp. and can attain heights as tall as 25–30 m, with a single main terminal shoot apex for linear growth. The date palm has a well-developed fibrous root system in which primary roots develop directly from the seeds/tree trunk with an average length of 4–6 m. The lateral roots originate from primary roots, which further produce lateral roots throughout their length. All date palm roots contain pneumatics, which act as respiratory organs. The plant contains pinnate leaves arranged alternately along the trunk. An adult date palm plant contains 100–125 leaves, with 40% juvenile, 10% fast-growing, and 50% photosynthetically active (Zaid and De Wet, 1999). The date palm is dioecious in nature, with separate trees producing male and female flowers in clusters called spadixes or spikes, within axils of leaves of the growth of previous years. Rarely, both pistillate and staminate flowers are produced on the same spike, and hermaphrodite flowers have also been reported in the inflorescence (Mason, 1915; Milne, 1918), although in very few cases. The staminate flowers are sweet-scented and generally have six stamens, each composed of two little pollen sacs. The stamens are surrounded by three waxy sepals and petals. The female flowers contain rudimentary stamens and are tricarpellate, consisting of three carpels that are closely pressed together and surrounded by a short perianth with a superior ovary (Figure 1). Pollination occurs by wind, or artificially, by dusting pollen grains collected from male spikelets onto female inflorescences. The fruit normally develops after fertilization from one carpel, which develops faster, while the other two carpels degenerate and drop later. The development of seeded fruit follows a sigmoidal curve with four distinct ripening stages: kimri, khalal (also know as bisir/bisr), rutab, and tamer stages (the names being of Arabic origin), which represent immature green, mature full-colored, soft brown, and hard raisin-like stages, respectively, containing average moisture content of 80%, 60%, 40%, and 20%, respectively (Fayadh and Al-Showiman, 1990; Al-Shahib and Marshall, 2003). The date fruit varies in size and shape depending on the cultivar and environment. With advancement in the developmental stages of the fruit, antioxidant activity increases until bisir and then decreases (Awad et al., 2011; Mohamed Lemine et al., 2014), whereas sugar content increases with ripening in the date palm fruit (Al-Mssallem et al., 2013) (Figure 1).

## Distribution and biodiversity

The date palm is one of the earliest cultivated fruit trees, with records of its cultivation in the areas of the Euphrates and Nile rivers going back to 3700 BC; while in Iran, Egypt, and Pakistan, the earliest records go back 7,000 years (Munier, 1973). The exact center of origin of the date palm is not certain; however, it is believed to have originated from the modern Iraqi region of Mesopotamia (Wrigley, 1995). Interestingly, the oldest seeds of palm, dating back to 5110 BC and 4670 BC, were reported from an island of Abu Dhabi known as Dalma (Rhouma et al., 2010). One of two wild species, *Phoenix reclinata* Jacq. from tropical Africa, or *P. sylvestris* (L.) Roxb. from India, or a hybrid of these two, is believed to be the progenitor of the date palm. However, many researchers believe that the tree originates from the Mesopotamian-Arabian Gulf area (Zohary and Hopf, 2000; Tengberg, 2012) and was later introduced into North Africa. However, genomic studies suggest that the genotypes from North Africa and the Middle East are genetically distinct, with higher genetic diversity in the North African date palm population (Hazzouri et al., 2015). Along with archaeological records, the population structure suggests two probable domestication sources in the date palm, one from the Middle East and the other from North Africa, forming two separate gene pools that diverged before domestication (Hazzouri et al., 2015; Zehdi-Azouzi et al., 2015). Also, the presence of admixed genotypes suggests that gene flow occurred between populations of eastern and western origins, primarily from east to west, as a result of human-mediated dispersal of the species after domestication (Zehdi-Azouzi et al., 2015). Whole-genome sequencing of wild and cultivated date palms reveals a complex domestication history with the contribution of at least two wild sources to the African cultivated palms (Gros-Balthazard et al., 2017). The date palm is thought to have spread globally in two directions: one from Mesopotamia to Iran, India, and Pakistan, and the other from Egypt toward Libya and the countries of the Maghreb and Sahel (Racchi and Camussi, 2018). The date palm is now more abundant in the arid regions of the Old World than in the temperate regions of the New World, with the most date palm trees found in Middle Eastern countries (Iraq, Iran, Saudi Arabia, United Arab Emirates, Oman, Yemen, etc.), followed by Africa (Algeria, Egypt, Libya, Mali, Morocco, Mauritania, Niger, Somalia, Sudan, Chad, Tunisia, etc.) (FAOSTAT, 2019). Apart from the Middle East and Africa, with the expansion of Islam the date palm has also been introduced into the United States and Europe (Chao and Krueger, 2007; Rivera et al., 2013).

The genus *Phoenix* consists of 12 closely-related species, making them cross-compatible for natural hybridization (Moore, 1963; Munier, 1973). Several natural hybrids were obtained from different countries: *P*. *dactylifera* × *P*. *sylvestris* (India), *P*. *dactylifera* × *P*. *canariensis* (Morocco, Algeria, and Israel), and *P*. *dactylifera* × *P*. *reclinata* (Senegal).

The date palm is generally diploid in nature, with 2n = 36 chromosomes (Darlington and Wylie, 1956), although polyploidy has been reported in Iraqi varieties (*n* = 64) (Panga, 2014). Also, differences in chromosome numbers have been observed between varieties Sayer (2n = 32) and Khasab (2n = 36). Al Salih et al. (1987) reported 2n = 32, 34, 36, and 64 date palm chromosomes. It would be interesting to investigate the crossability among accessions with different ploidy levels for creating newer genetic variations.

## Molecular marker-assisted genetic diversity in the date palm

Genetic diversity refers to the genetic variability present within species, subspecies, cultivars, or populations, and can be measured at the morphological, physiological, biochemical, or molecular levels. A total of 3,000–5,000 date palm cultivars exist globally. The cultivar diversity and production indices across major date palm growing countries are presented in Table 1 (Abul-Soad et al., 2015; Al-Yahyai and Al-Khanjari, 2008; Al-Yahyai and Khan, 2015; Ba-Angood, 2015; Bashah, 1996; Battaglia et al., 2015; Bouguedoura et al., 2015; Elshibli, 2009; Hajian and Hamidi-Esfahani, 2015; Mahar, 2007; Osman, 1984; Rabei et al., 2012; Sedra, 2015; Zabar and Borowy, 2012; Zaid and De Wet, 1999; FAOSTAT, 2020). Despite collections of traditionally cultivated genotypes, duplications have also been reported among them. Therefore, approximately only 10% of the total cultivars existing globally are believed to be unique and commercially important (Johnson, 2011). The commercialization of preferred date palm cultivars prompted an increase in area of cultivation, thereby enhancing the practice of monoculture, which ultimately led to a significant decline in genetic (or species) diversity in the date palm. Characterizing, conserving, and using date palm collections globally is one of the felt needs that can be effectively met using high-density genomics approaches. In recent years, initiatives aimed at characterizing large collections of gene banks have succeeded. As an example, 100,000 wheat and 30,000 maize accessions of the gene bank of the International Maize and Wheat Improvement Center (CIMMYT) were characterized (Seeds of Discovery: Unlocking the genetic potential of maize and wheat). This sort of approach could provide a suitable option for characterizing date palm genetic resources globally.

Research into developing biochemical and molecular markers for date palms began in the late 1970s, and was later used for genetic diversity analysis. Various isozyme markers were used to study the inheritance of multiple traits in date palm seedlings (Torres and Tisserat, 1980) and genetic diversity (Bennaceur et al., 1991), or to develop a cultivar identification system (Bendiab et al., 1998) for date palms. Advances in molecular biology, and the development of a PCR-based marker system during the 1990s led to studies using various molecular marker systems, either individually or in combination, to unravel the genetic diversity and phylogenetics of the date palm. Initially, DNA-based marker systems, such as RFLP and RAPD, were used to identify polymorphic markers associated with date palm cultivars (Corniquel and Mercier, 1994). Even though RAPD markers have low reproducibility, they have been used to study the genetic diversity in various date palm accessions in different countries (Table 2). Other types of dominant multi-locus markers, such as ISSR markers on their own or in combination with RAPD markers, have also been used in genetic diversity analysis of the date palm (Table 2). Genetic diversity analyses using RAPD or ISSR markers, or a combination of both, have shown that huge genetic similarity (more than 90%) exists among various date palm genotypes. A comparison between four female date palm trees and four unknown male trees of the Egyptian date palm, using RAPD markers, shows that genetic similarity existed not only between female cultivars (87.5%–98.9%), but also between unknown male trees (88.9%–95.3%) (Soliman et al., 2003). A more reproducible multi-locus marker, AFLP, has been used either alone, or in combination with other marker systems, to assess genetic variations present in date palm cultivars across the globe. Initially, an attempt was made to develop a genetic map using AFLP markers with a population derived from Um-Assla and KL-96 (El-Kharbotly et al., 1998). Later, either AFLP or fluorescently-labeled AFLP primers were used to study genetic diversity among various accessions of the date palm, with genetic similarity ranging from approximately 10%–75% (Cao and Chao, 2002; Devanand and Chao, 2003a, 2003b; El-Khishin et al., 2003; El-Assar et al., 2005; Jubrael et al., 2005; Elhoumaizi et al., 2006; Rhouma et al., 2007; Khierallah H. et al., 2011, Khierallah et al., 2011 H. S.). The AFLP markers were also used to assess intra-varietal differences (El-Assar et al., 2005; Elhoumaizi et al., 2006), and to study the genetic fidelity of plants raised *in vitro* (Diaz et al., 2003; Al Kaabi et al., 2007). Apart from being used to study diversity, these multi-locus markers have also been used for cultivar identification (Table 2) (Corniquel and Mercier 1994; Al-Moshileh et al., 2004; Sabir J. S. et al., 2014).

**TABLE 2:** Application of biochemical and molecular markers in genetic diversity studies of the date palm.

Markers type	Markers used	Genotypes studied	Geographical location of studied genotypes	Study type	Citation
Isozyme	5	26 female and 20 male date palm cultivars and breeding populations	California	Inheritance in date palm seedlings	Torres and Tisserat (1980)
Isozyme	7	186 plants belonging to 31 cultivars	Algeria	Genetic diversity analysis	Bennaceur et al. (1991)
Isozyme	3	28 genotypes	Morocco	Varietal identification	Bendiab et al. (1998)
RFLP and RAPD	-	5	-	Cultivar identification	Corniquel and Mercier (1994)
RAPD	19	43	Morocco, Iraq, Tunisia	Genetic diversity	Sedra et al. (1998)
ISSR	12	18	Tunisia	Genetic diversity	Zehdi et al. (2002)
ISSR	15	8	Ethiopia	Genetic diversity	Takele et al. (2021)
RAPD		3 female and 4 male trees	Egypt	Genetic diversity	Soliman et al. (2003)
RAPD	37	13	Saudi Arabia	Genetic diversity	Al-Khalifah and Askari (2003)
RAPD	12	5	Saudi Arabia	Genetic diversity and cultivar identification	Al-Moshileh et al. (2004)
RAMPO	18	30 female and 10 male trees	Tunisia	Genetic diversity	Rhouma et al. (2008)
RAPD	3	10	Bahrain	Genetic diversity	Pathak and Hamzah (2008)
RAPD and ISSR	5 each	4	Saudi Arabia	Genetic diversity	Abdulla and Gamal (2010)
ISSR and AFLP	13 and 6	10	Saudi Arabia	Genetic diversity and cultivar identification	Sabir et al. (2014a)
SSR	22	24 female and 6 male trees	Iraq	Genetic diversity	Khierallah et al. (2011b)
AFLP	6	11 female and 7 male trees	Iraq	Genetic diversity	Khierallah et al. (2011a)
RAPD and ISSR	35 and 15	18 female and 5 male trees	Syria	Genetic diversity	Haider et al. (2012)
RAPD and ISSR	30 and 12	10 female and 7 male trees	Iraq	Genetic diversity	Khierallah et al. (2014)
RAMPO and AFLP	18 and 6	40	Tunisia	Genetic diversity	Soumaya et al. (2011)
ISSR and DAMD	5 and 8	9	UAE	Genetic diversity	Purayil et al. (2018)
ISSR	10	14	Iran	Genetic variability and population structure	Sharifi et al. (2018)
cpDNA sequences	-	47	Iran	Genetic diversity	Sharifi et al. (2018)
RAPD	3	10	Bahrain		Pathak and Hamzah (2008)
RAPD and ISSR	35 and 15	18	Syria	Genetic diversity	Haider et al. (2012)
RAPD	19	43	Morocco, Iraq, Tunisia	Genetic diversity and cultivar identification	Sedra et al. (1998)
RAPD	5	10	Nigeria	Genetic diversity	Emoghene et al. (2015)
RAPD and ISSR	27 and 21	20	Algeria	Genetic diversity	Guettouchi et al. (2017)
IR fluorescence- labeled AFLP markers	4	21	USDA germplasm collection	Genetic diversity	Cao and Chao (2002)
IR fluorescence-labeled AFLP markers	-	2	California (United States)	Genetic purity testing of Deglet Noor and Medjool	Devanand and Chao (2003a, 2003b)
Fluorescence-labeled AFLP	4	Various accessions of Medjool and Deglet Noor	Morocco, Egypt, and California (United States)	Genetic similarity/diversity within accession	Elhoumaizi et al. (2006)
AFLP	4	47 accessions of Medjool and Deglet Noor	Egypt	Genetic similarity/diversity within accession	El-Assar et al. (2005)
AFLP	5	5 individuals of 3 genotypes		Varietal identity among offshoots	Diaz et al. (2003)
AFLP	4	18	Iraq	Genetic relationship and varietal identification	Jubrael et al. (2005)
AFLP	-	10	UAE	Genetic fidelity of tissue culture‒raised plants	Al Kaabi et al. (2007)
AFLP	6	40	Tunisia	Genetic diversity	Rhouma et al. (2007)
SCoT (start codon targeted)	4	113 trees of 13 varieties	Iran	Genetic diversity	Saboori et al. (2020)
SSR	14	49	Tunisia	Genetic diversity	Zehdi et al. (2004)
SSR	16	37 female and 23 male trees	Sudan and Morocco	Genetic diversity	Elshibli and Korpelainen (2008)
SSR	10	200 individuals from 19 populations	Sudan	Genetic diversity	Elshibli and Korpelainen (2009)
SSR	5	26	Tunisia	Genetic diversity	Hammadi et al. (2011)
SSR	14	74 female and 27 male trees	Tunisia	Genetic diversity	Zehdi et al. (2012)
SSR	17	31	Algeria	Genetic diversity	Akkak et al. (2009)
SSR	37	18	-	Varietal identification	Johnson et al. (2009)
SSR	10	21	Oman, Bahrain, Iraq, and Morocco	Genetic diversity in tissue culture‒raised plants	Al-Ruqaishi et al. (2008)
SSR	10	15	Qatar	Genetic diversity	Ahmed and Al-Qaradawi (2010)
SSR		11	Qatar	Genetic diversity	Elmeer et al. (2011)
SSR	14	59 plants from 12 cultivars	Qatar	Inter- and intra-varietal genetic diversity	Elmeer and Mattat (2015)
SSR	22	16	Iraq, Iran, and Africa	Genetic diversity	Arabnezhad et al. (2012)
SSR	16	377 female trees of 18 cultivars and 63 male trees	Libya	Molecular typing and diversity analysis	Racchi et al. (2014)
SSR	15	200 trees consisting of 191 females belonging to 26 cultivars, and 9 male trees	Morocco	Genetic diversity	Bodian et al. (2012)
SSR	16	377 trees belonging to 18 cultivars	Libya	Genetic diversity	Racchi et al. (2014)
SSR	10	89 female plants from 18 cultivars	Sudan	Genetic diversity	Elsafy et al. (2016)
SSR	46	45	Pakistan	Genetic diversity	Faqir et al. (2016)
SSR	22	32	Saudi Arabia	Genetic diversity	Al-Faifi et al. (2016)
Fluorescence-labeled SSRs	17	82	Australia	Genetic diversity	Al-Najm et al. (2017)
SSR	255	1,066 date palms from 411 cultivars	12 different countries	Genetic diversity	Salomon-Torres et al. (2017)
SSR	18	113 date palms involving 31 males and 82 females	Nigeria	Genetic diversity	Zango et al. (2017)
SSR and chloroplast mini-satellite	18 and 1	414 trees belonging to 114 varieties	Algeria	Genetic diversity	Moussouni et al. (2017)
SSR	12	38 wild date palm genotypes	Bangladesh	Genetic diversity	Huda et al. (2019)
SSR	8	24 male pollinizers	Tunisia	Genetic diversity	El Kadri et al. (2019)
Mitochondrial and plastid genome-based SNPs	-	9 varieties		Molecular characterization	Sabir et al. (2014b)
GBS-based SNPs	-	70 female genotypes and four other species			Mathew et al. (2015)
SNPs	-	62 cultivars		Genetic diversity and gene-trait relationship	Hazzouri et al. (2015)
GBS-based SNPs	-	191 cultivars		Genetic diversity	Thareja et al. (2018)
*MatK, rbcl, atpB,* and SNPs	-			SNP typing and varietal identification	Al-Dous et al. (2011)
ITSs	-	15	Tunisia	Haplotype identification and diversity analysis	Maina et al. (2019)

Because of their abundance and dispersion throughout the genome, their co-dominance nature, ease of usage, and ability to automate, microsatellites or simple sequence repeats (SSR) have proven an ideal choice for cultivar identification and genetic diversity analysis, as well as for linkage and QTL mapping, and marker-assisted breeding. Billotte et al. (2004) made the first attempt to develop SSR markers for date palms using a (GA)_n_-enriched library. They further designed 16 SSR primers, and tested their amplification on 40 DNA samples of *P. dactylifera* from various origins, as well as on 11 other species of *Phoenix*. Later, several attempts were made to identify and develop SSR markers for date palms using a genomic DNA library enriched for microsatellite sequences (Akkak et al., 2009; Arabnezhad et al., 2012; Al-Faifi et al., 2016). Apart from using microsatellite-enriched libraries, available sequence information, such as ESTs, have been used to develop EST-SSRs as well as gene-based SSRs, and they have been characterized with their functional annotations (Zhao et al., 2012, 2017). With the availability of various draft assemblies of date palm genomes, "genome sequence information has been used to develop 1090 SSRmarkers (Hamwieh et al., 2010). Using the draft genome sequence of the date palm, Mokhtar et al. (2016) identified 172,075 SSR motifs, with a frequency of 450.97 SSRs per Mb. A total of 130,014 SSRs (75.6%) were located within the intergenic regions, while 42,061 SSRs (24.4%) were located in the genic regions. Furthermore, 111,403 SSR primer pairs were designed, with a density of 291.9 SSR primers per Mb.

Numerous genetic diversity analyses have been conducted with the help of microsatellite markers in different countries: Tunisia, Qatar, Libya, Morocco, Sudan, Pakistan, Saudi Arabia, Niger, Algeria, Sudan, the United States (California), Australia, etc. (Zehdi et al., 2004, 2012; Al-Ruqaishi et al., 2008; Elshibli and Korpelainen, 2008, 2009; Akkak et al., 2009; Ahmed and Al-Qaradawi, 2010; Hamwieh et al., 2010; Hammadi et al., 2011; Bodian et al., 2014; Racchi et al., 2014; Elmeer and Mattat, 2015; Al-Faifi et al., 2016; Elsafy et al., 2016; Faqir et al., 2016; Al-Najm et al., 2017; Moussouni et al., 2017; Zango et al., 2017; El Kadri et al., 2019) (Table 2).

Salomon-Torres et al. (2017) reviewed the performance of 255 SSR markers for studying diversity among 1,066 date palm plants from 411 cultivars in 12 countries, and recommended a set of 19 SSR markers as useful for further genetic diversity analysis. Recently, studies have looked into the genetic diversity of worldwide date palm germplasm accessions, using SSRs (Chaluvadi et al., 2014; Zehdi-Azouzi et al., 2015; Salomon-Torres et al., 2017), SNPs (Hazzouri et al., 2015; Mathew et al., 2015), or comparisons of whole genomes (Hazzouri et al., 2015). Through sequencing of 62 varieties of date palms from 12 countries, Hazzouri et al. (2015) show that Middle Eastern genotypes form a separate group from North African genotypes, with North African genotypes having higher nucleotide diversity than Middle Eastern/South Asian genotypes. Similar results were obtained by Mathew et al. (2015), where the authors used the sequence data from 70 date palm accessions. Such accessions are mostly propagated through tissue culture; however, variations among accessions with the same name suggest that somaclonal mutation is ongoing during the process of subculturing during tissue culture. Under some circumstances, huge genetic variations within the same accession suggest that, since the date palm is dioecious in nature, there is a probability of random crossing events in addition to the controlled outcrossing, and the plants might have been raised from the seeds, resulting in an increase in the genetic distance between the genotypes.

Apart from their use in genetic diversity analysis, SSR markers have been used in developing a molecular identification key, as well as in molecular-typing for identification of the characterized cultivars (Zehdi et al., 2012; Racchi et al., 2014). These SSR markers, developed and characterized across various date palm accessions, can further be used for identifying candidate genes and understanding the genetic basis of traits of interest, which may further help in molecular breeding for the genetic improvement of the date palm tree.

### Single nucleotide polymorphisms

As the third generation of molecular markers, single nucleotide polymorphisms (SNPs) are more stable, and have higher conformity of inheritance than other marker systems (Gupta et al., 2001). With the whole-genome sequencing of the date palm, the first attempt to identify SNPs was carried out by Al-Dous et al. (2011), where researchers called 1,748,109 SNPs in 381 Mb of sequence, yielding a heterozygosity rate of 0.46%, or 1 SNP/217 bp, but the distribution of the SNPs was skewed, with 49% of the SNPs within every 50 bp. Sabir J. S. M. et al. (2014) used the mitochondrial and plastid genome sequences of nine date palm varieties to examine SNPs, but found a low level of variation, suggesting the preferred use of nuclear SNPs for molecular characterization of date palm cultivars. A genotyping-by-sequencing (GBS) approach was used to identify 13,000–65000 SNPs comparing the genomes of 70 female cultivars from different date palm growing regions and four other *Phoenix* species (Mathew et al., 2015). Based on the whole-genome re-sequencing of 62 cultivars, a catalog of approximately 7 million SNPs in date palms was developed (Hazzouri et al., 2015). Recently, the GBS approach was followed by using re-sequenced data of 191 date palm cultivars to identify SNPs and assess the genetic diversity among the date palm trees grown in Qatar. This study revealed that these trees in Qatar are of eastern origin and their genetic diversity does not associate with different regions (Thareja et al., 2018). Faqir et al. (2019) sequenced maturase K (matK), ribulose biphosphate carboxylase larger subunit (*rbcL*), the ATP synthase subunit b (atpB) gene of the chloroplast genome, and 12 DNA fragments from the nuclear genome of seven cultivars. Based on the sequenced data, the researchers identified unique SNP signatures and developed an SNP-typing system for varietal identification of date palm cultivars from Pakistan.

The internal transcribed spacer (ITS) sequences of 15 Tunisian date palm accessions were compared to identify four haplotypes, and the haplotypic and nucleotide diversities were found to be low among the studied genotypes (Maina et al., 2019). Further phylogenetic analysis revealed that the Tunisian populations of date palm evolved under a neutral model, and a demographic equilibrium seems to be maintained within the studied genotypes.

## Trait-specific markers in the date palm

Most of the molecular studies of the date palm have been carried out for genetic diversity and phylogenetic analysis, as well as for cultivar identification, with limited progress made in developing trait-specific molecular markers. Most efforts have been aimed at identifying markers associated with Bayoud disease resistance, or for sex determination. *Fusarium oxysporum* f. sp. *albedensis* causes Bayoud disease, which is one of the most devastating of all diseases in date palm trees (Michielse and Rep, 2009; El Modafar, 2010).

Bendiab et al. (1992) carried out isozyme polymorphism analysis using esterase (EST), glutamate oxaloacetate transaminase (GOT), endopeptidase (ENP), and alcohol dehydrogenase (ADH) polymorphisms in different F_1_ populations derived from seven female cultivars crossed with two males (Table 3). They found out three loci viz., Got2, Est 1, and Enp that could be used for hybrid screening. Benslimane et al. (1994, 1996) isolated two mitochondrial-like plasmid DNA (S and R plasmids) sharing 99% sequence similarity, except for 109 bp of sequence that was present in only the S plasmid. The S plasmid was found in Bayoud-susceptible genotypes, whereas the R plasmid was found in Bayoud-resistant Moroccan genotypes. Later, employing a PCR-based approach on 36 date palm varieties, Quenzar et al. (2001) confirmed the study of Benslimane et al. (1994, 1996), and reported that the simultaneous presence of the R plasmid and absence of the S plasmid can be considered a reliable marker for Bayoud resistance (Table 3). Salem et al. (2007) used this plasmid-based analysis system to check the susceptibility of Mauritanian date palm cultivars to Bayoud disease. Furthermore, using progenies of two controlled crosses, the authors showed that Bayoud strictly follows maternal transmission as controlled by the mitochondrial genome. The R and S mitochondrial plasmids have been used for molecular characterization of date palm cultivars from Algeria (Guettouchi et al., 2017), Syria (Haider and Nabulsi, 2012), and Saudi Arabia (Saleh et al., 2015).

**TABLE 3:** Trait-specific markers in date palms.

Trait	Marker	References
Bayoud disease	Biochemical: esterase (EST-1), glutamate oxaloacetate transaminase (GOT-2), endopeptidase (ENP)	Bendiab et al. (1992)
	R and S mitochondrial plasmid	Benslimane et al. (1994, 1996)
Brittle leaf disease	Double-stranded chloroplast RNA	Namsi et al. (2006, 2007); Triki et al. (2003)
Gender-specific	Biochemical: peroxidase and glutamate oxaloacetate activity higher in females	Bekheet et al. (2008); Qacif et al. (2007)
	RAPD: OPA10-490, OPA12-750, and OPD10-800 specific to females and OPA12-370 and OPD10-675 specific to males	Younis et al. (2008)
	ISSR: HB10-1010, HB9-340, HB12-375, 814-590, and 844A-920 specific for males	
	RAPD-derived SCAR marker	Dhawan et al. (2013)
	ISSR: IS_A02 (390) specific to female plants and IS_A71 (380bp) specific to male plants only	Al-Ameri et al. (2016b)
	SCoT-derived SCAR marker of size 253 bp specific to male trees	Al-Ameri et al. (2016a)
	RAPD-derived SCAR marker	Al-Qurainy et al. (2018)
	SRY gene-specific marker for identification of male plants	Mohei et al. (2019)
	SSRs: mPdIRDP80, mPdIRDP50, mPdIRDP52, mpdCIR48, and DP-168	Cherif et al. (2013); Elmeer and Mattat (2012); Maryam Jaskani et al. (2016)
	SNPs	Al-Dous et al. (2011)

Brittle leaf disease, known as *maladie des feuilles cassantes* in French, was first observed in southern Tunisia (Djerbi, 1983). It later spread to reach epidemic levels by 1986. The exact causal pathogen is not yet determined; however, the symptom of the disease is associated with manganese deficiency and the presence of a small double-stranded chloroplast RNA (Triki et al., 2003; Namsi et al., 2006, 2007; Marqués et al., 2008). Namsi et al. (2006) used chloroplast RNA, and developed a digoxigenin (DIG)-labeled probe for early diagnosis of BLD, which consistently gave positive hybridization signals, irrespective of cultivars, the severity of symptoms, or the geographic location (Table 3).

The date palm is a dioecious plant, and the sex of the plants can be determined only at the time of flowering, which takes 5–7 years (Shaheen, 1990). If the sex of the plants could be determined at the early seedling stage, this could save resources and time, as farmers need many female plants and only a few superior male plants for pollination. Hence, maintaining a proper male:female ratio is of the utmost importance for better production in the field. Sex determination at the early seedling stage is thus one of the major requisites for establishing commercial date palm orchards. Therefore, the identification of markers linked to the sex of plants is of key importance for date palm cultivation. For the first time, Siljak-Yakovlev et al. (1996) developed a cytological method in which staining with chromomycin shows the presence of an extra heterochromatin region on both the arms of the male chromosome, which was considered sex determinant. Atia et al. (2017) describe cytological-based markers to distinguish date palm sex through localization of 45S and 5S rDNA markers on date palm chromosomes using the fluorescence *in situ* hybridization (FISH) technique.

A few biochemical markers, such as peroxidase and glutamate oxaloacetate, reportedly differentiate between male and female date palms, with a differential response of peroxidase and glutamate oxaloacetate activity observed in female plants versus male plants (Qacif et al., 2007; Bekheet et al., 2008). Over the past 2 decades, several attempts have been made to understand the genetic basis of sex determination in date palms using various types of DNA markers, such as RFLP, RAPD, ISSR, and SSRs. Using RAPD primers, several polymorphic markers have been identified with the potential to distinguish male from female plants among different cultivars (Ben et al., 2000; Soliman et al., 2003; Bekheet et al., 2008).

Younis et al. (2008) used a combination of RAPD and ISSR techniques to identify three fragments derived from RAPD markers specific to females (OPA10-490, OPA12-750, OPD10-800), and two for males in RAPD analysis (OPA12-370, OPD10-675), as well as five specific markers for males through ISSR analysis (HB10-1010, HB9-340, HB12-375, 814-590, 844A-920) (Table 3). However, in the past decade, attempts have been made to develop SCAR (sequence-characterized amplified region) markers for sex determination in date palms. The genomic DNA of 10 male genotypes of unknown origin and 10 female genotypes were pooled in equal quantities separately, and 100 RAPD primers and 104 ISSR primers were used to identify sex-specific markers. One of the RAPD primers, OPA-02, amplified an ≈1.0-kb fragment specifically in pooled as well as individual samples of male genotypes, and was later converted into a SCAR marker, which amplified a fragment of 406 bp in both female and male genotypes, and a unique fragment of 354 bp only in male genotypes (Dhawan et al., 2013). The developed SCAR marker was further validated in 25 female and 10 male date palms belonging to different varieties collected from different locations. Later, using an ISSR marker, Al-Ameri et al. (2016b) identified a 390-bp fragment from the amplicons of primer IS_A02, specifically in a female plant, and a 380-bp fragment from the amplicons of primer IS_A71, specifically in male plants only. These fragments were sequenced further to develop sequence-specific markers. Al-Ameri et al. (2016a) developed a SCAR marker of size 253 bp, specific to male trees based on cDNA fingerprinting of start codon targeted (SCoT) marker, and validated it independently on male and female trees. Al-Qurainy et al. (2018) developed a SCAR marker linked to sex-specific regions in the genome of the date palm using RAPD marker OPC-06, which was producing a band of 186 bp in male plants only. Recently, a gene, *SRY1*, involved in initiating sex determination, was identified on the Y chromosome of the date palm, and was tested with 100% efficiency for identifying male plants at the seedling stage (Mohei et al., 2019). Apart from RAPD and ISSR markers, a few microsatellite markers (e.g., mPdIRDP80, mPdIRDP50, mPdIRDP52, mpdCIR48, and DP-168) possessing the capacity for sex differentiation in the date palm have also been identified (Elmeer and Mattat, 2012; Cherif et al., 2013; Maryam Jaskani et al., 2016). Al-Dous et al. (2011) identified a region harboring 1,605 SNPs linked to sex through *de novo* genome sequencing, and proposed that the date palm follows an XY system of gender inheritance (Table 3). A 6-Mb region has been further mapped onto the distal end of chromosome 12, which has been found to be associated with sex determination (Hazzouri et al., 2019). Recently, Torres et al. (2021) identified 16-bp male-specific sequences in the date palm Y chromosome.

Date palms are facing a severe threat around the globe from red palm weevil (*Rhynchophorus ferrugineus* Olivier). So far, no molecular marker has been reported that deciphers resistance to this dreaded date palm pest. Using a historic long-term ongoing field trial with 18 date palm varieties, researchers at the International Center for Biosaline Agriculture have identified the pattern of preference/sensitivity and non-preference/tolerance (anti-xenosis behavior) for red palm weevil of specific date palm varieties. They are further trying to understand if there could be a robust molecular/genetic basis of RPW resistance in the date palm, and further to identify the molecular markers linked to this RPW resistance. The developed markers will not only help in selecting resistant genotypes, but will also help in developing genotypes with RPW resistance through accelerated molecular breeding.

## Date palm genomics

**FIGURE 2:**
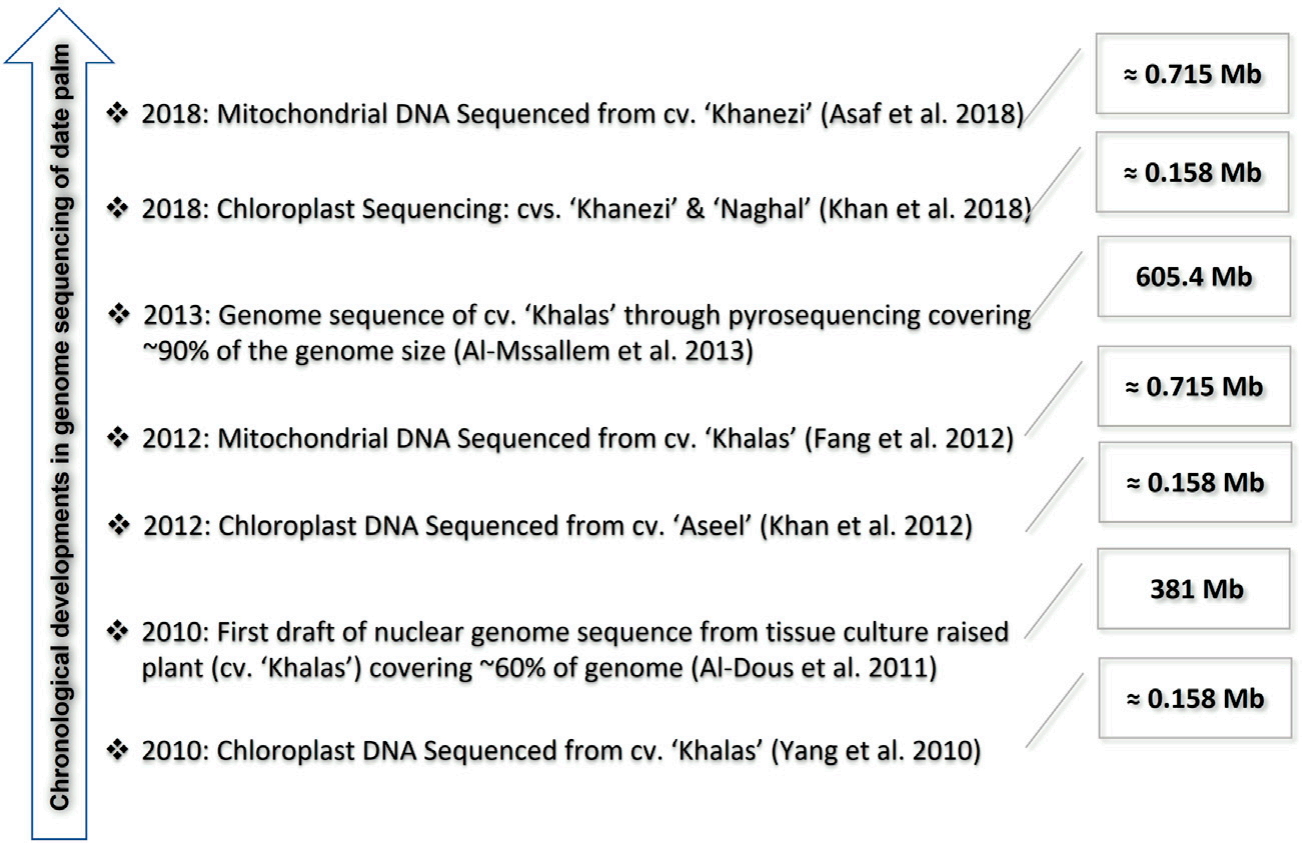
Advances in genome sequencing of the date palm.

Genomics deals with the sequencing and analysis of the structure of the genome of an organism, predicting the genes, and their locations and functions in the genome. Initially, the date palm genome was considered to be relatively smaller than 250 Mb, with 41% of the region consisting of genes, and the remaining genome considered a non-coding region (Barakat et al., 1999). However, it was later found that the size of the date palm genome ranged from 550 to 650 Mbp (Malek, 2010). Initially, a random genomic library of Tunisian date palm varieties was constructed from total cellular DNA, and amplified using RAPD markers. The library consisted of inserts from 200 to 1,600 bp and was supposed to have potential application for generating probes for molecular characterization of date palm varieties through southern hybridization. Al-Faifi et al. (2017) generated 6,943 high-quality ESTs from a normalized cDNA library of the date palm cultivar, Sukkari. The generated ESTs were assembled into 6,362 unigenes and were further functionally annotated. The first genetic map of the date palm cv. Khalas was developed by Mathew et al. (2014), using ∼4,000 SNPs spanning a total of 1,293 cM. Furthermore, the analysis suggested that the telomeric region on linkage group 12 may be the sex-determination region of the date palm. A total of 19% of the draft genome sequence scaffolds were placed onto the linkage groups, and the analysis results showed that approximately 1.9 cM represents 1 Mb on the map (Mathew et al., 2014). The chronological developments in genome sequencing of date palm is given in Figure 2.

### Organellar genomes

With the advances in next-generation sequencing (NGS) technologies during the past decade, progress in the genomics of the date palm has been made at an unprecedented pace. The complete chloroplast genome of the date palm cultivar Khalas was sequenced using pyrosequencing and was found to be of 158,462 bp in size, consisting of 112 unique genes and 19 duplicated fragments in the inverted repeat (IR) regions, and arranged in a typical quadripartite structure (Yang et al., 2010). Furthermore, 78 SNPs located in genes with vital functions were identified with potential for detecting intra-varietal polymorphisms within a date palm population. Using a combination of Sanger-based and next-generation sequencing strategies, Khan et al. (2012) sequenced the complete date palm chloroplast genome from the Pakistani cultivar Aseel. The size of the genome was found to be 158,458 bp, consisting of a large single-copy (LSC) region of 86,195 bp, and a small single-copy (SSC) region of 17,711 bp, separated by an IR region of 27,276 bp. The chloroplast genome consisted of 138 genes, of which 89 were protein-coding, 39 were tRNA, and 8 were rRNA genes. Furthermore, a comparison of the Khalas and Aseel chloroplast genome led to the identification of SNPs and mono-nucleotide SSRs. Recently, Khan et al. (2018) sequenced the chloroplast genome of two economically important date palm cultivars, Khanezi and Naghal, using the Illumina HiSeq4000 sequencing platform. The chloroplast genome sizes of Naghal and Khanezi were 158,210 bp and 158,211 bp, respectively, consisting of 138 genes. The phylogenetic analysis based on the whole chloroplast genome and 68 shared genes of four cultivars (Khanezi, Naghal, Khalas, and Aseel) yielded identical phylogenetic trees, with Khanezi and Naghal forming single clades with cultivars Khalas and Aseel, respectively.

Fang et al. (2012) published the first mitochondrial genome of the date palm cv. Khalas. The genome assembly consisted of 715,001 bp encoding 38 proteins, 30 tRNAs, and 3 ribosomal RNAs. The protein-coding sequence consists of only 6.5% (46,770 bp) of the mitochondrial genome, whereas the rest of the genome sequence (93.5%) was found to comprise chloroplast-derived (10.3%) and non-coding sequences. Recently, the mitochondrial genome of *P. dactylifera* var. Khanezi, consisting of 715,120 bp, was published (Asaf et al., 2018). The mitochondrial genome consisted of 67 genes encoding 24 transfer RNAs, 3 ribosomal RNAs, and 40 protein-coding genes. Apart from these two mitochondrial genomes, another unpublished assembly is available in GenBank from an unknown cultivar (MG257490.1), consisting of 585,493 bp (Figure 2).

### Nuclear reference genomes

Whole-genome sequencing is fundamental for understanding the molecular basis of complex traits for crop improvement. With the rapid progress in NGS technology and the simultaneous availability of bioinformatics tools, the past decade has seen unprecedented developments in date palm genomics, leading to the development of two draft genome sequences and genetic maps. The first attempt to develop the draft genome sequence of the date palm cv. Khalas was made by Al-Dous et al. (2011). The genome was sequenced from tissue culture‒raised plants using the Illumina platform. Unfortunately, it covered only ∼60% of the genome and consisted of 380 Mb of sequence, spanning mainly gene-rich regions, including 25,059 gene models. However, this reference genome was found to be highly fragmented, with about 60,000 scaffolds showing a median length of ∼30 kb. These authors further identified a genomic region linked to the sex of the plant, and provided evidence that the date palm follows an XY system of gender inheritance. Subsequently, using pyrosequencing, Al-Mssallem et al. (2013) reported another genome assembly of higher quality from the same date palm cultivar Khalas. This genome assembly has a total length of 605.4 Mb, covering more than 90% of the genome and 96% of the genes. They further built a larger pool of gene models, consisting of 41,660 models with a total of 42,957 isoforms in 10,363 scaffolds. The sequenced genome analysis demonstrated genome-wide duplication after either ancient whole-genome duplications or massive segmental duplications. Genetic diversity analysis showed that the stress resistance and sugar metabolism-related genes are enriched in the chromosomal regions where the density of SNPs is relatively low. Scrutiny of the late embryogenesis abundant (LEA) gene family revealed that group 2 LEA genes are specifically abundant in date palms, with 62 group 2 LEA members showing generally ubiquitous expression, whereas LEA1, LEA3, LEA4, LEA5, LEA6, seed maturation protein, and dehydrin were found to be either seed or male flower associated. This date palm draft genome assembly has also been included in the reference sequence (RefSeq) collection in the National Center for Biotechnology Information (NCBI), and gene models have been included in UniProtKB and the Kyoto Encyclopedia of Genes and Genomes (KEGG) databases for further exploration. In 2019, Hazzouri et al. (2019) released a new date palm draft genome (“BC4 male”). This draft genome spanned 772 Mb and was assembled into 2,390 scaffolds (Figure 2).

### Functional genomics of the date palm

The first attempt to gain insight into carbon partitioning, comparative transcriptome, and metabolome analysis in oil palm and date palm mesocarp led to the identification of several sugars and fatty acid metabolism genes/transporters involved in fatty acid and sugar accumulation in date and oil palm, respectively (Bourgis et al., 2011). Yin et al. (2012) carried out cDNA sequencing of the date palm fruits of Khalas at seven different developmental stages and identified 10 core cell division genes, 18 ripening-related genes, and 7 starch metabolic enzymes, which are involved in nutrition storage and sugar/starch metabolism. To generate and annotate the gene model of the date palm, Zhang et al. (2012) carried out in-depth transcriptomic sequencing from different tissues and at several developmental stages, and generated 30,854 annotated gene models from the cultivar Khalas. These were further assigned to Gene Ontology and KEGG pathways for future research aimed to unravel the genetic regulatory networks governing organ development and differentiation in the date palm (*P. dactylifera*)*.* Whole-genome transcriptome analysis of eight tissues (root, seed, bud, fruit, green leaf, yellow leaf, female flower, and male flower), using the Roche/454 GS FLX platform, showed higher gene expression levels in developing tissues, such as male and female flower, root, and bud, than in the four other tissues, due to the need for more energy than in the relatively mature tissues (Fang et al., 2012). To identify the differentially expressed genes (DEGs) involved in fruit development and ripening, Al-Mssallem et al. (2013) carried out transcriptome analysis at seven distinct fruit developmental stages (0, 15, 30, 60, 90, 120, and 135 days post-pollination), and identified 4,134 DEGs whose expression varies significantly among the seven fruit developmental stages. The enrichment analysis of DEGs revealed that most of the molecular events involved in biological regulation, transcription, and regulation of RNA metabolic processes are down-regulated in the late stage of fruit development, whereas events involved in sugar accumulation, such as gluconeogenesis, cellular carbohydrate metabolism, and small molecule biosynthesis were up-regulated, resulting in unusually high sugar content in the dates. Hazzouri et al. (2019) carried out RNA-Seq analysis in date palm fruit at different developmental stages. The results indicated that the expression of alkaline/neutral invertase (*A/N-INV1*) was maximum at ≈105 days after pollination, whereas the expression of *cell wall invertase* (*CWINV1* and *CWINV3*) genes peaked at 120 days after pollination, showing their positive role in sugar accumulation during fruit development. Recently, Naganeeswaran et al. (2020) performed transcriptome assembly from the embryogenic calli of the date palm cultivar Khalas, and reported 63,888 Gene Ontology (GO) terms and 122 small RNAs that were annotated from the assembly (Table 4).

**TABLE 4:** Transcriptomic studies in date palm.

Study type	Focused trait studies	References
Transcriptome and metabolome	Carbon partitioning, sugars, and fatty acid metabolism	Bourgis et al. (2011)
cDNA sequencing	Cell division genes, ripening-related genes, and sugar/starch metabolism	Yin et al. (2012)
Transcriptome	30,854 annotated gene model and Gene Ontology and KEGG pathways assignment. Gene networks controlling organ development	Zhang et al. (2012)
Transcriptome	Differential expression of genes involved in energy metabolism in different tissues	Fang et al. (2012)
Transcriptome	Genes involved in fruit development and ripening	Al-Mssallem et al. (2013)
Transcriptome	Genes and small RNAs expressed in embryogenic calli	Naganeeswaran et al. (2020)
Transcriptome	Genes/pathways involved in imparting salinity tolerance	Radwan et al. (2015)
Transcriptome	Salinity-responsive small RNA libraries from roots and leaves	Yaish et al. (2015)
Transcriptome	Salinity-responsive genes in roots and leaves	Yaish et al. (2017)
Transcriptome and metabolome	Genes and metabolites in response to mild heat, drought, and combination of both stresses	Safronov et al. (2017)
Transcriptome	Genes involved in detoxifying cadmium toxicity	Rekik et al. (2019)
Suppression-subtractive hybridization	Genes involved in BLD tolerance	Saidi et al. (2010)
RT-PCR	Differential expression of genes in response to BLD in roots and leaves	Saidi et al. (2012)
Transcriptome	Differentially expressed genes in response to RPW infestation	Giovino et al. (2015)

Date palms generally grow under adverse climatic conditions and have therefore developed stress tolerance during their evolution. The date palm can survive under extreme drought, heat, and relatively high soil salinity (Yaish and Kumar, 2015), thereby providing a valuable genome source for mining abiotic stress tolerance genes. However, limited research work has been carried out to identify and exploit the abiotic stress-responsive genes from the date palm. To understand the molecular mechanisms underlying salinity tolerance in the date palm, Radwan et al. (2015) undertook salinity-responsive transcriptome analysis in young roots of the date palm cv. Deglet Beida, which led to the identification of 1939 differentially expressed genes involved in tolerance of salt stress. RNA-Seq analysis further revealed that salinity stress activates abscisic acid signaling pathways through SNF1-related protein kinase 2, and several key genes involved in sodium uptake and transport were found to be down-regulated, thereby slowing down uptake and transportation in plant tissues under stress conditions. Yaish et al. (2015) generated salinity-responsive small RNA libraries from leaves and roots of date palm seedlings. Deep sequencing using Illumina Hiseq2000 led to the identification of 153 homologs of conserved miRNAs, 89 miRNA variants, and 180 putative novel miRNAs from the date palm plant. Differential expression analysis revealed that 57 miRNAs in leaves and 27 miRNAs in roots were significantly regulated in response to salinity, whereas 12 miRNAs were commonly regulated in both leaves and roots. The targets of the identified miRNAs were the genes with known functions in plant salt tolerance, such as potassium channel AKT2-like proteins, vacuolar protein sorting-associated protein, calcium-dependent protein, and mitogen-activated proteins. Later, expression profiling in the leaves and roots of date palm seedlings revealed 194 differentially expressed transcripts in both leaf and root tissue in response to salinity stress (Yaish et al., 2017). Gene ontology analysis revealed that metabolic pathways, such as photosynthesis, sucrose and starch metabolism, and oxidative phosphorylation were enriched in leaves, whereas genes involved in membrane transport; phenylpropanoid biosynthesis; purine, thiamine, and tryptophan metabolism; and Casparian strip development, were enriched in roots in response to salinity stress. Salinity-responsive genes, such as putative potassium transporter 8, abscisic acid receptor PYR1 and 4, indole-3-acetic acid-amido synthetase GH3, along with a pyrophosphate-energized vacuolar membrane proton pump, were commonly induced in both roots and leaves. Using transcriptomic and metabolomic profiling, Safronov et al. (2017) studied the adaptation mechanism in the date palm toward mild heat, drought, and the combination of both. The results showed an increase in soluble carbohydrates, such as fructose and glucose derivatives, suggesting a switch to carbohydrate metabolism and cell wall biogenesis in response to these stresses. Increased transcriptional activation of genes involved in reactive oxygen species production occurred in response to all three treatments (drought, heat, and combined heat and drought). By contrast, under heat and combined heat and drought stress, genes enriched for circadian and diurnal rhythm motifs were differentially expressed, suggesting a stress avoidance mechanism in response to these stresses (Safronov et al., 2017). Another group of researchers employed salinity-responsive whole-genome bisulfite sequencing and mRNA sequencing in the roots of date palms (Al-Harrasi et al., 2018). The bisulfite sequencing revealed that the methylated regions increased in response to salinity, specifically at mCHG and mCHH sequences. However, when researchers correlated gene expression with DNA methylation, they observed that DNA methylation was not the primary agent that controls gene expression under salinity conditions (Al-Harrasi et al., 2018). Overexpressing the cDNA library of the date palm in *Saccharomyces cerevisiae*, and screening on a synthetic minimal medium containing 1.0 M of NaCl, resulted in the identification of genes such as aquaporins (*PIP*), serine/threonine protein kinases (*STKs*), ethylene-responsive transcription factor 1 (*ERF1*), and peroxidases (*PRX*) with potential salt-tolerance functions (Patankar et al., 2018). Rekik et al. (2019), through transcriptome analysis in leaves of *Phoenix dactylifera* cv. Deglet Nour, proposed a glutathione pathway involved in detoxifying cadmium under Cd stress conditions, and further identified genes encoding heavy metal transporters and chelators in response to heavy metal stress. Patankar et al. (2019b) isolated aquaporin genes (*PdPIP1;2*) and characterized their role in response to drought and salinity tolerance by overexpressing them in yeast and *Arabidopsis*. The overexpression of an aquaporin gene in yeast resulted in improved oxidative stress tolerance, whereas overexpression in *Arabidopsis* resulted in increased salinity and drought tolerance with increased biomass, chlorophyll content, and root length in transgenic plants (Patankar et al., 2019a). Further, Patankar et al. (2019a) isolated metallothionein 2A (*PdMT2A*) and characterized its role in abiotic stress tolerance in yeast and *Arabidopsis*. The transformed yeast cells have shown tolerance against drought, salinity, and oxidative stresses. The *Arabidopsis* plants overexpressing the metallothionein 2A (*PdMT2A*) gene have shown tolerance against salinity by maintaining a high K^+^/Na^+^ ratio, and against drought and oxidative stress (Patankar et al., 2019b). Al-Harrasi et al. (2020) isolated a salt-inducible vascular highway 1-interacting kinase (*PdVIK*) and characterized its role in response to various abiotic stresses through heterologous overexpression in yeast and *Arabidopsis*. Jana and Yaish (2020, 2021) isolated and characterized the glyoxalase-I gene (*PdGLX1*) and glyoxalase III genes (*PdDJ-1*) for their roles in mitigation of abiotic stress tolerance through overexpression in bacterial and yeast systems. This study further suggested that *PdGLX1* and *PdDJ-1* genes play an important role in methylglyoxal detoxification and in maintaining reactive oxygen species balance under stress conditions in date palms.

Apart from understanding the transcriptional response of abiotic stress tolerance and fruit development, a couple of studies have been carried out to identify the genes involved in biotic stress tolerance. To understand the molecular mechanisms involved in the BLD of the date palm, Saidi et al. (2010) constructed suppression-subtractive cDNA libraries from BLD-affected and non-affected trees and identified the genes that were up-regulated in response to BLD. The genes associated with stress response, metabolism, protein synthesis, and signal transduction were found to be specifically up-regulated in BLD-affected trees. Later, through RT-PCR analysis, Saidi et al. (2012) showed that the transcripts of MnSOD decreased in affected leaves and roots, unlike the transcripts of FeSOD and Cu/Zn-SOD, whose expression increased in these tissues, revealing that BLD decreases the expression of manganese-related genes in date palm trees. To understand the molecular basis of red palm weevil (*Rhynchophorus ferrugineus* Olivier) resistance in *Phoenix canariensis*, Giovino et al. (2015) carried out deep transcriptome analysis in leaves of healthy and infested trees at two stages (middle and late infestation) and identified 54 genes that were differentially regulated during the middle stage in response to RPW infestation (Table 3). Further enrichment analysis showed that phenylpropanoid-related pathways were induced during the middle infestation period.

### Resequencing of the date palm

With the availability of genetic maps, organellar and nuclear reference genomes of the date palm, several research groups carried out whole-genome resequencing of date palms to identify QTLs and SNP markers as well as to study date palm diversity and phylogenetic history. Hazzouri et al. (2015) resequenced 61 female date palm accessions and 1 male (cv. Fard4), and detected 7,176,238 SNPs at a rate of ∼12 SNPs per kb. Genome-wide scans for selection suggested that there were ∼36 genomic regions in the genotypes of the Middle East, and 20 genomic regions in North African genotypes associated with positive selection which may underlie the geographic adaptation of these genotypes in these areas. They further characterized candidate mutations in the genes of the pathways associated with key agronomic traits, such as disease resistance, fruit ripening, fruit color, flowering time, and sugar metabolism. Hazzouri et al. (2015) further suggested that the R2R3 myb-like *virescens (VIR)* gene controls fruit color in the date palm. The varieties with red fruit color were found to have an intact *VIR* gene in the homozygous state, whereas the varieties with yellow fruit color had a *copia*-like retrotransposon insertion in the *VIR* gene in either the homozygous or heterozygous state. Using the GBS approach on 70 female cultivars from different date palm growing regions and four other *Phoenix* species, Mathew et al. (2015) showed that there are two centers of earliest cultivation and that the date palm is indigenous to North Africa. Whole-genome sequencing of several wild and cultivated date palms revealed a complex domestication history of date palm trees involving the contribution of a wild relative during the spread of cultivation from their original domestication center in the Arabian Gulf to North Africa (Gros-Balthazard et al., 2017). Sequence analysis of more than 200 mitochondrial and chloroplast genomes from a geographically diverse set of date palms showed that the most common cultivated date palms contain four haplotypes associated with the geographic region of cultivar origin (Mohamoud et al., 2019). Recently, Hazzouri et al. (2019) carried out genome-wide association studies of the sex-determining region, and of 21 fruit traits. GWAS analysis resulted in the identification of the R2R3-MYB transcription factor (*VIR* gene) associated with fruit color. The authors further identified an ≈1.1-Mb region consisting of invertase genes that were found to be associated with sugar composition in date palm fruit (Table 5).

**TABLE 5:** Whole-genome resequencing studies in date palms and their wild relatives.

References	Number of germplasm/accessions resequenced	Key findings
Al-Dous et al. (2011)	5 female and 3 male and one F_1_ progeny	• First draft genome assembly of the date palm (cv. Khalas)
		• Identified 3,518,029 SNPs
		• Identified XY sex-determination model and region controlling sex on XY chromosomes
Al-Mssallem et al. (2013)	3 female and 1 male	• Improved genome assembly of the date palm (cv. Khalas)
		• Functional analysis of genes involved in abiotic stress tolerance and genes involved in sugar metabolism during fruit ripening
Hazzouri et al. (2015)	61 female and 1 male	• Genetic diversity analysis among the cultivars from North Africa and the Middle East
		• Candidate mutations for trait variation in genes involved in the pathways for key agronomic traits
		• *Virescens* (*VIR*) gene encoding R2R3 myb-like transcription factor was found to be associated with fruit color variation
Gros-Balthazard et al. (2017)	2 date palm cultivars	• Discovered wild date palm populations in remote Oman
	3 wild date palms	• Studied population structure and diversity analysis in the date palm
	1 *Phoenix sylvestris*	• Revealed complex domestication history of date palms
	1 *Phoenix atlantica*	
Torres et al. (2018)	15 female and 13 male trees representing all 14 *Phoenix* species	• Identified male-specific sequences
		• Further identified CYP703 and GPAT3 genes involved in male flower development in the date palm
Hazzouri et al. (2019)	145 female and 12 male	• Improved genome assembly for *P. dactylifera*
		• Genome-wide association studies of the sex-determining region and fruit traits
		• Confirmed previous finding that fruit color is controlled by *VIRESCENS* gene
		• Identified *invertase* genes controlling sugar composition in date palms

## Genomic databases for date palm

During the past decade, several attempts have been made to sequence and re-sequence the several date palm genotypes, leading to the accumulation of a huge amount of genomic data. Further, several SSR and SNP markers have been developed. However, this information is scattered across research publications. This necessitates the development of genomic databases for the date palm so that the developed genomic information can be used more efficiently. The first attempt at this was by Mokhtar et al. (2016), who established a Date Palm Molecular Markers Database (DPMMD) providing useful genomic information (http://dpmmd.easyomics.org/). This database contains information on more than 3,611,400 DNA markers involving SSRs and SNPs, genetic linkage maps, KEGG maps, DNA-barcode, as well as all previously published date palm articles in PubMed-indexed journals from 1976 to 2017. Apart from this, the DRDB (Date Palm Resequence Database) was developed by CAS Key Laboratory of Genome Sciences and Information and Joint Center of Excellence in Genomics, King Abdulaziz City for Science and Technology (He et al., 2017). This database consists of information about 6.3 million SNPs and 246,000 SSRs from 62 date palm cultivars. Apart from these two, there is no concise database for date palm genomics.

## Summary and way forward

**FIGURE 3:**
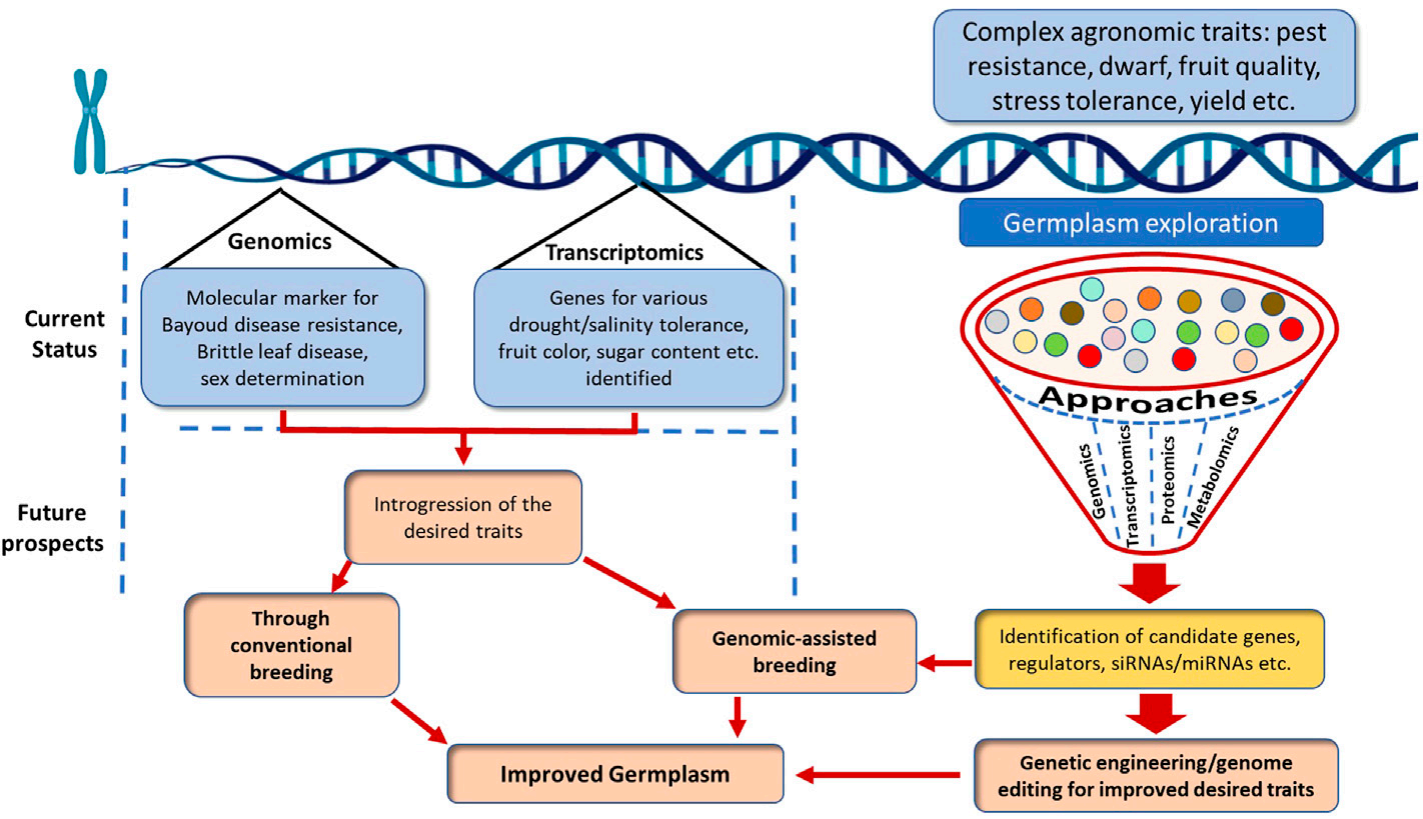
Current status and prospects in date palm improvement.

The date palm has immense regional relevance but requires global attention, as not many advanced research laboratories outside the Middle East and North Africa are giving due attention to date palm genomics. Although limited genomic studies of the date palm over the last decade have led to the identification of a couple of key genes associated with fruit color and sugar accumulation, this is still a long way from what is needed to unravel the hidden mysteries of this tree. Despite the huge existing diversity within the date palm genus, there is little understanding of the genetic factors underlying various biotic and abiotic stresses. The robustness and reliability of a marker are central to its usefulness in a genetic improvement program. Several breeder-friendly molecular markers, such as SSRs and SNPs, have been identified, but the extent to which these markers explain variation still needs to be validated on a large scale. Several abiotic stress responsive genes, and genes associated with fruit traits, have been identified. However, the identified genes/QTLs need to be introgressed in date palm improvement programs, either through breeding or genetic engineering. The use of genetic engineering tools for genome editing is the need of the moment, at least for game-changing traits such as the genetic mechanism of red palm weevil resistance, but this is still lagging because of limited concerted efforts with this crop. Further, studies on the role of small RNAs (siRNA and miRNA) are lacking. It is time to obtain feedback from stakeholders on desired traits in the different genetic backgrounds, and to generate foundational knowledge from diverse research disciplines, including genomics. An extensive germplasm exploration is required for the desired trait combinations ranging from plant architecture and stress tolerance to fruit yield and quality. A concerted effort is therefore needed, employing genomics, transcriptomics, proteomics and metabolomics for identification of candidate genes/genomic regions associated with complex agronomic traits, which can then be further introgressed in popular date palm cultivars/accessions, either through genetic engineering/editing or conventional breeding. An efficient ideotype breeding strategy for the desired date palm variety will be helpful for its improvement (Figure 3). In sum, there should be consortium- or mission-mode-based collaborative efforts to generate and use genomic information in breeding, genetic engineering, or genome editing research for developing new farmer-friendly date palm varieties.
